# Exploring heart rate variability in polycystic ovary syndrome: implications for cardiovascular health: a systematic review and meta-analysis

**DOI:** 10.1186/s13643-024-02617-x

**Published:** 2024-07-24

**Authors:** Seyedeh Tarlan Mirzohreh, Padideh Panahi, Fariba Heidari

**Affiliations:** 1 Faculty of Medicine, Tabriz University of Medical Sciences, Tabriz, Iran; 2 Department of Community and Family Medicine, Faculty of Medicine, Tabriz University of Medical Sciences, Tabriz, Iran

## Abstract

**Objectives:**

Polycystic ovary syndrome (PCOS) is a prevalent and complex endocrine disorder that affects women of reproductive age. It has significant implications for female endocrine function, reproductive health, and metabolic disturbances, including insulin resistance, impaired glucose tolerance, and dyslipidemia. Studies have shown that decreased heart rate variability (HRV), a marker of autonomic dysfunction, is associated with adverse cardiovascular events. Recent research has focused on investigating autonomic function in PCOS, and some studies have suggested altered autonomic drive in these patients. The aim of this systematic review and meta-analysis was to evaluate cardiac autonomic function by analyzing HRV in women with PCOS.

**Methods:**

This systematic review was prepared using PRISMA reporting guidelines. The databases searched were PubMed, Scopus, Web of Science, and Cochrane. Risk of Bias was assessed using ROBINS-I for non-RCTs. The GRADE approach was employed to evaluate the level of certainty in the evidence for each outcome. In order to identify the underlying cause of high heterogeneity, a subgroup analysis was conducted. Sensitivity analysis was checked. A random effect model was used and calculated a pooled standardized mean difference (SMD) with a 95% confidence interval (CI).

**Results:**

Seventeen articles were included in the final analysis, varied in quality, ranging from a "low" to a "high risk of bias". Combined analyses indicated a notable decrease in HRV among individuals with PCOS compared to the control group. Significant changes were observed in SDNN (SMD: -0.763, 95%CI [-1.289 to -0.237], *p*=0.004), PNN50 (SMD: -1.245, 95%CI [-2.07, -0.419], *p*=0.003), LF/HF ratio (SMD: 0.670, 95%CI [0.248, 1.091], *p*=0.002), HFnu (SMD: -0.873, 95%CI [-1.257, -0.489], *p*=0.000), LFnu (SMD: 0.840, 95%CI [0.428, 1.251], *p*=0.000) and TP (SMD: -1.997, 95%CI [-3.306, -0.687], *p*=0.003). The heterogeneity was partially explained by types of study design. Subgroup analysis revealed significant alterations of HRV in normal-weighted and overweight PCOS cases. Conversely, no significant changes in HRV were observed in obese PCOS cases.

**Conclusion:**

The findings of this meta-analysis provide evidence suggesting diminished HRV in individuals with PCOS compared to non-PCOS control group.

**Supplementary Information:**

The online version contains supplementary material available at 10.1186/s13643-024-02617-x.

## Introduction

Polycystic ovary syndrome (PCOS) is a prevalent endocrine disorder that has a significant impact on a woman's overall health. Its effects are not limited to the reproductive age and can have long-term consequences [1]. Initially, PCOS was identified as a combination of anovulation and hyperandrogenism. However, it is now understood to be a different manifestation of metabolic syndrome [2]. Apart from the reproductive abnormalities, about two-thirds of women with PCOS also experience metabolic dysfunction [3]. This metabolic dysfunction increases their susceptibility to developing cardiovascular risk factors, including insulin resistance [4], type 2 diabetes mellitus [5], coronary heart disease [6], atherogenic dyslipidemia [7], and cerebrovascular morbidity [8]. Recent studies have even demonstrated impaired cardiovascular functional capacity in individuals with PCOS [9]. It is worth noting that heart rate is influenced by multiple physiological systems and mechanisms, such as autonomic neural activity, respiratory function, and the endocrine system [10].

Heart rate variability (HRV) is a noninvasive, reproducible, and statistical measure of the cyclic beat-to-beat variation in heart rate, which correlates with individual autonomic activity and is used to assess risk in a wide variety of both cardiac and noncardiac disorders [11]. A higher risk of cardiovascular diseases is positively correlated with lower variability, whereas good cardiac adaptability is indicated by high variability [12]. The presence of cardiac autonomic imbalance is commonly observed in individuals with cardiometabolic disorders such as diabetes, hypertension, and metabolic syndrome [13, 14]. Insulin resistance, hyperandrogenism, increased BMI, vascular alterations, and inflammatory processes are closely associated with heightened sympathetic tone and reduced HRV [15, 16]. In women with PCOS, the risks associated with these factors are even more pronounced as they contribute to a detrimental cycle involving androgen production and adipogenesis [17, 18]. Furthermore, excessive androgens in women play a role in stimulating and exacerbating insulin resistance [19].

The evaluation of HRV in PCOS has yielded conflicting findings in previous research. While some surveys have reported no significant changes in HRV measures in PCOS [20–22], a number of studies have presented evidence of cardiac autonomic dysfunction associated with PCOS [23–25]. This systematic review and meta-analysis aimed to provide a comprehensive examination to identify potential patterns or discrepancies in HRV featuring central autonomic function among PCOS individuals, and contribute valuable insights to further our understanding of the interplay between PCOS and cardiac autonomic regulation. The rationale behind investigating central autonomic function in PCOS stems from the growing understanding that PCOS is not solely confined to reproductive and metabolic aspects but may also involve dysregulation of neural control.

## Materials and methods

This systematic review and meta-analysis follows the Preferred Reporting Items for Systematic Reviews and Meta-Analyses (PRISMA) guidelines [26]. The PRISMA checklist is provided as supplement (S1-document). The protocol for this work was registered in the International Prospective Register of Systematic Reviews (PROSPERO) (identifier: CRD42022340798).

### Eligibility criteria

To be considered for inclusion, published studies had to meet the following criteria: (1) studies employed valid research designs with clearly defined methodology, (2) studies identified PCOS cases using either of the PCOS diagnostic criteria, including the Rotterdam, National Institutes of Health (NIH), World Health Organization (WHO) and Androgen Excess PCOS (AEPCOS) criteria, (3) studies enrolled on young women aged 18 and above, diagnosed with PCOS, (4) studies reported at least one HRV index, measuring either time-domain or frequency-domain HRV measures, (5) studies reported its data using a valid statistical reporting format, (6) studies involved appropriately matched participants serving as the control group and assessed the relevant parameters both in PCOS cases and the control group, (7) studies involved women in their reproductive age with or without PCOS, (8) studies excluded individuals with known cardiovascular disease, thyroid disease, neoplasms, pregnancy or breast-feeding, smoking, chronic alcohol consumption, diabetes mellitus, hypertension and renal impairment.

The overall exclusion criteria for the meta-analysis were as follows: (1) studies reported in the form of abstracts, case reports, case series, reviews, editorials and practice guidelines, (2) studies involving women in their menopausal or postmenopausal stage with and without PCOS, (3) studies assessing HRV in cases only diagnosed with metabolic syndrome and not specifically with PCOS, and (4) studies measuring autonomic function with means of a method other than HRV (e.g., Muscle Sympathetic Nerve Activity (MSNA)).

### Information sources

A thorough search was conducted in the PubMed, Scopus, Web of Science, and Cochrane databases to locate relevant studies published until August 2022. Additionally, a manual search of the reference lists of the identified articles was carried out.

### Search strategy

The search strategy of Scopus was conducted as follows: ((TITLE-ABS-KEY (parasympath* OR sympath* OR sympathovagal OR vagal OR vagus OR "autonom* nerv*" OR "ans" OR "pns" OR "sns" ) ) OR (ALL ("heart rate variability" OR "cycle length variability" OR "r-r variability" OR "hrv" OR "heart rate recovery" ) ) ) AND (TITLE-ABS-KEY ("polycystic ovar* syndrome" OR "polycystic ovar* disease" OR "stein leventhal syndrome" OR "pcos" OR "sclerocystic ovar*" ) ). The search strategy employed for PubMed, Web of Science, and the Cochrane Library was similar to that used for Scopus and its table is provided as supplement **(S2-document)**. Furthermore, two reviewers independently reviewed the reference lists of systematic reviews and selected studies to ensure that all pertinent articles were included in the analysis.

### Study selection

Two reviewers independently assessed each title and abstract, and if the articles fulfilled the inclusion criteria, the full text was reviewed. The eligibility of the selected articles was then assessed by the same two reviewers through an evaluation of their full texts. Any discrepancies were resolved through discussion with a third reviewer. The study selection process was summarized using the PRISMA flow diagram.

### Data extraction

Following the extraction of data, the information was gathered through Microsoft Excel spreadsheets. The subsequent dataset comprises: study characteristics (study design, year of publication, and first author), type of PCOS diagnostic criteria, number of individuals in each study population (PCOS cases and matched controls) and HRV measures (time-domain and frequency-domain indices). Potential confounding factors were carefully considered to ensure the robustness of the study findings. These factors included participants' age, BMI, blood pressure, heart rate, lipid profile, sex hormones profile, and study designs. To address the influence of these confounders, relevant data were extracted from the included studies.

### Definitions of outcomes

#### Time-domain measures

Mean RR: Normal-to-Normal average RR interval.

SDNN: The standard deviation of normal-to-normal intervals.

SDANN: The standard deviation of the 5-minute average NN interval.

RMSSD: The root mean square of successive interval differences.

PNN50: The percentage of successive intervals that differ by more than 50 ms from adjacent NN intervals.

NN50: The number of pairs of successive intervals that differ by more than 50 ms from adjacent NN intervals.

#### Frequency-domain measures

LF band: The absolute power of the low-frequency band with a frequency of 0.04–0.15 Hz.

LFnu band: The relative power of the low-frequency band with a frequency of 0.04–0.15 Hz in normal units.

HF band: The absolute power of the high-frequency band with a frequency of 0.15–0.4 Hz.

HFnu band: The relative power of the high-frequency band with a frequency of 0.15–0.4 Hz in normal units.

LF/HF: The ratio of LF to HF band.

TP: The total power corresponds to the sum of the four spectral bands, LF, HF, ULF (ultralow frequency) and VLF (very low frequency).

RMSSD and pNN50 are frequently employed time-domain metrics for evaluating parasympathetic nerve activity [27, 28]. Conversely, SDNN and TP measurements encompass both sympathetic and parasympathetic activities [29]. It is crucial to recognize that SDANN should not be regarded as a replacement for SDNN since it is derived from 5-minute segments rather than the entire 24-hour time series [30]. Additionally, research indicates that SDANN does not provide any supplementary valuable information [27].

HF and HFnu power indicate parasympathetic activity at the sinus node [31]. LF and LFnu primarily reflect a combination of sympathetic and parasympathetic activity. At low breathing rates, LF power predominantly represents parasympathetic activity, but under normal respiratory rates, this frequency index reflects baroreflex activity rather than cardiac sympathetic innervation [31, 32]. The LF/HF ratio is considered to represent the sympathovagal balance, with the sympathetic nervous system potentially contributing to LF power, while HF power is generated by the parasympathetic nervous system [33].

### Risk of *bias* assessment

ROBINS-I was employed to evaluate the methodological quality and risk of bias in the included studies, particularly focusing on HRV outcomes including both time domain and frequency domain measures. This tool encompasses the assessment of seven potential sources of bias, including confounding bias, bias in participant selection, bias in intervention classification, bias due to deviations from intended interventions, bias resulting from missing data, bias in outcome measurement, and bias in the selection of reported results [34]. Importantly, no studies were excluded based on the assessment of bias risk. The certainty of overall evidence was assessed using the Grading of Recommendations Assessment, Development and Evaluation (GRADE) method [35]. The assessment of evidence certainty for individual outcomes relied on five distinct criteria: (1) limitations of the study design; (2) consistency of results; (3) directness; (4) precision and (5) potential for publication bias. A decrement of one level in certainty was implemented for each unfulfilled criterion. The certainty of evidence for all HRV measures, including both time and frequency domain measures, was evaluated in line with the GRADE approach.

### Synthesis methods

The standardized mean differences (SMD) pooled the data, with 95% confidence intervals (CIs). Chi-square tests and I2 tests were used to analyze the interstudy statistical heterogeneity. To calculate the pooled effect, either fixed-effects or random-effects model was used according to the heterogeneity, study design and sample size. I2 values of 25%, 50%, and 75% were considered to represent low, moderate, and high levels of heterogeneity, respectively. If there was obvious heterogeneity, a subgroup meta-analysis was conducted to identify the underlying heterogeneity. Additionally, we performed a univariate meta-regression model to elucidate the influence of potential moderators. This model included baseline heart rate, BMI, and SBP as potential predictors. The implementation of the meta-regression test relied on the inclusion of at least ten studies in the meta-analysis. The availability of data on lipid profile and sex hormones profile was limited, thereby precluding their inclusion in the meta-analysis. The stability of the pooled results was evaluated through a sensitivity analysis using the "one study removed" method. Moreover, publication bias was assessed by visually inspecting funnel plots of SMD vs. standard error. When at least 10 studies were available for analysis, Begg's tests and Egger's tests were employed to evaluate the potential publication bias. If there was an obvious publication bias, a trim-and-fill analysis was used to determine the underlying origin of the publication bias. All analyses were conducted using Comprehensive Meta-Analysis Version 3. P-value< 0.05 was considered significant in all tests.

## Results

### Study Selection

**Fig. 1:**
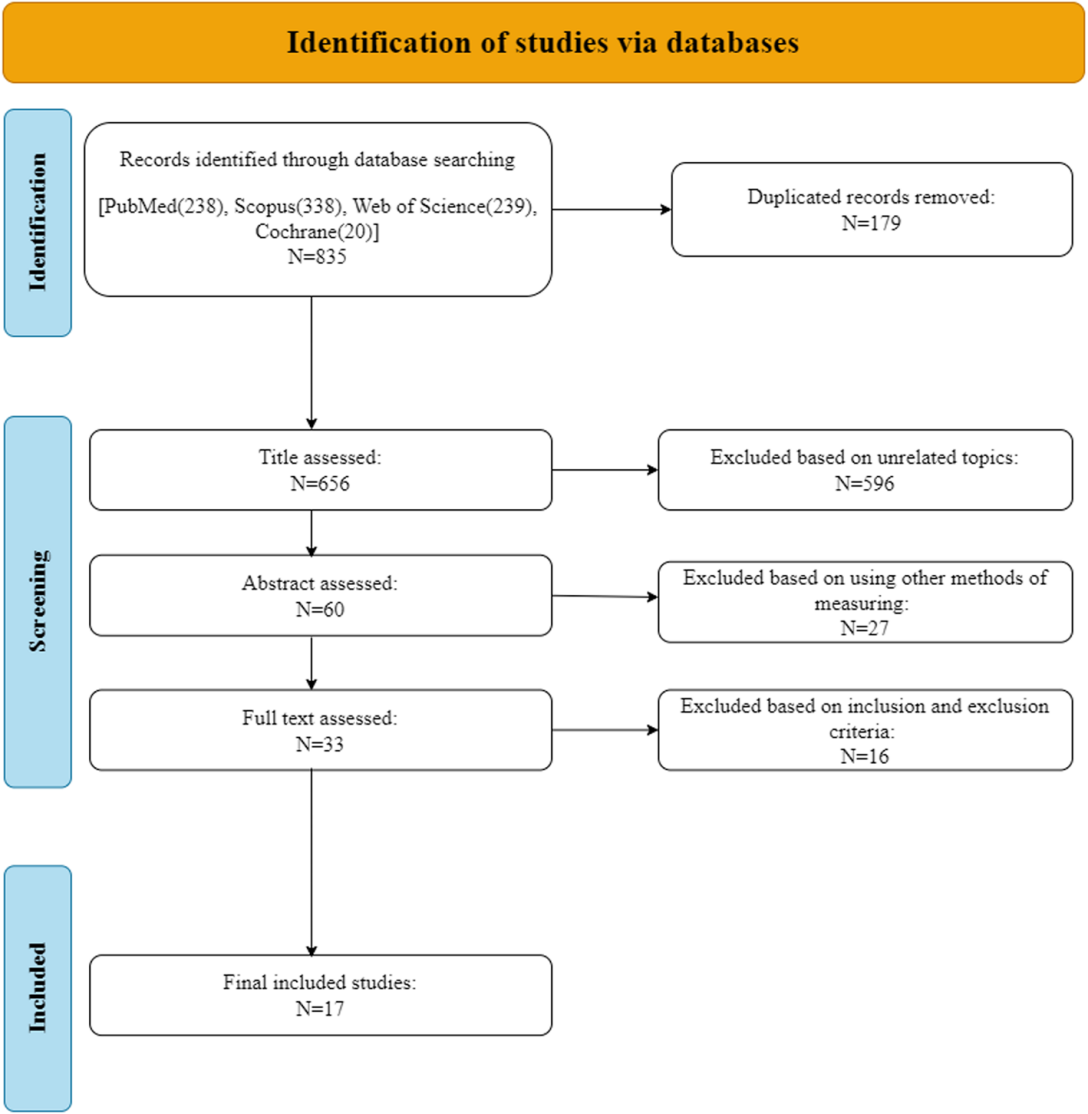
PRISMA Flow Diagram

The study flowchart is shown in Fig. 1; our search strategy revealed 835 studies. After removing duplications, 656 studies underwent title assessment. Of these, 60 studies were eligible for abstract review. After surveying abstracts, 33 studies met the inclusion and exclusion criteria and were perused for full text. Finally, 17 studies were qualified to be included in this systematic review and meta-analysis, and the rest did not meet the inclusion criteria; the reasons for their exclusions are provided in the supporting information section (S3-document).

### Study characteristics

Table 1 displays the characteristics of the included studies in the analysis. The investigation identified 17 studies, comprising 6 with a cross-sectional study design [24, 25, 36–39] and 11 employing a case-control approach [20–23, 40–46]. Among these, 4 studies (Di Domenico et al.[38], Hashim et al.[39], Lambert et al.[20], and Philbois et al.[46]) enrolled individuals with PCOS who were classified as obese with a body mass index (BMI) exceeding 30. The majority of the studies presented both time-domain and frequency-domain measures of HRV. Notably, two articles (Hashim et al.[39], and Özkeçeci et al.[21]) exclusively reported time-domain measures, while one study (Balamurugan et al.[40]) solely reported frequency-domain measures of HRV. In terms of country-specific analysis, India has been the subject of six studies [24, 36, 37, 40–42], while Turkey [21, 22, 44, 45] and Brazil [23, 38, 43, 46] have each been the focus of four studies. Additionally, there has been one study conducted in Iraq [39] and another in Austria [20]. Majority of the investigations included anthropometric features such as age, BMI, systolic blood pressure (SBP), diastolic blood pressure (DBP), and baseline heart rate of the participants. Two studies (Hashim et al.[39], and Philbois et al.[46]) assessed two distinct groups of women with PCOS. For the purpose of analysis, each group was treated separately, with the non-obese PCOS cases marked as number 1 and the obese cases as number 2 (e.g., Philbois et al. 2019 (1) and Philbois et al. 2019 (2)).

**Table 1:** Study characteristics

**First author and year of publication**	**Study design**	**Country**	**Publication language**	**PCOS diagnosis criteria**	**BMI Status**	**No. PCOS**	**No. Controls**	**Cardiovascular parameters**	**Anthropometric features**	**Major Findings**
Yildirir et al, 2006 [45]	Case- control	Turkey	English	NIH	Overweight	30	30	HF, LF, HFnu, LFnu, LF/HF ratio	Age, BMI, Baseline heart rate, SBP, DBP	Significant altered HRV measures in PCOS cases.
Tekin et al, 2008 [44]	Case- control	Turkey	English	Rotterdam (classic type)	-	26	24	RMSSD, SDNN, SDANN, pNN50, TP, HF, LF	Age, Baseline heart rate, SBP	Significant alterations in some diagnostic and prognostic parameters gleaned from a standard treadmill exercise test and HRV analysis.
Di Domenico et al, 2013 [38]	Cross-sectional	Brazil	English	Rotterdam	Obese	30	23	RMSSD, Mean-RR, PNN50, LFnu, HFnu, LF/HF	Age, BMI, SBP, DBP	1) Impaired autonomic modulation in response to sympathetic stimulation. 2) Negative and significant correlation between total testosterone levels and frequency domain HRV indices during stress in PCOS.
Saranya et al, 2014 [37]	Cross-sectional	India	English	Rotterdam	Overweight	31	30	RMSSD, SDNN, pNN50, NN50, mean-RR, TP, HF, LF, HFnu, LFnu, LF/HF ratio	Age, BMI, Baseline heart rate, SBP, DBP	1) Significant alterations of HRV measures in PCOS cases. 2) Significant correlation between LF/HF ratio and BMI, WHR, heart rate.
Kuppusamy et al, 2015 [36]	Cross-sectional	Inida	English	Rotterdam	Overweight	35	32	RMSSD, SDNN, pNN50, NN50, mean-RR, TP, HFnu, LFnu, LF/HF ratio	Age, BMI, Baseline heart rate, SBP, DBP	1) Significant alterations of HRV measures in PCOS cases. 2) Significant correlation between LF/HF ratio and BMI, heart rate and IR.
Hashim et al, 2015 (1) [39]	Cross-sectional	Iraq	English	Rotterdam	-	32	20	RMSSD, SDNN, SDANN, pNN50	SBP, DBP	No significant changes of HRV measures in PCOS cases.
Hashim et al, 2015 (2) [39]	Cross-sectional	Iraq	English	Rotterdam	Obese	32	20	RMSSD, SDNN, SDANN, pNN50	BMI, SBP, DBP	1) Significant alterations of HRV measures in PCOS cases. 2) Negative correlation between SDNN and WHR.
Balamurugan et al, 2015 [41]	Case- control	India	English	Rotterdam	Normal	24	24	RMSSD, SDNN, NN50, pNN50, mean-RR, HF, LF, HFnu, LFnu, LF/HF ratio	Age, BMI, SBP, DBP, Baseline heart rate	Altered cardiac autonomic activity and unfavorable metabolic profile in PCOS cases.
Lambert et al, 2015 [20]	Case- control	Austria	English	Rotterdam	Obese	19	21	RMSSD, SDNN, mean-RR, HFnu, LFnu, LF/HF ratio	Age, BMI, Baseline heart rate, SBP, DBP	No significant changes of HRV measures in PCOS cases.
Balamurugan et al, 2016 [40]	Case- control	India	English	WHO	Normal	24	24	TP	BMI, SBP, DBP, Baseline heart rate	Decreased total variability of heart rate in lean and ideal weight PCOS.
Özkeçeci et al, 2016 [21]	Case- control	Turkey	English	NIH	Normal	23	25	RMSSD, SDNN, SDANN	Age, BMI, Baseline heart rate, SBP, DBP	No significant changes of HRV measures in PCOS cases.
Ribeiro et al, 2016 [23]	Case- control	Brazil	English	Rotterdam	Overweight	27	26	Mean-RR, HF, LF, HFnu, LFnu, LF/HF ratio	BMI, Baseline heart rate, SBP, DBP	1) Altered cardiac autonomic activity in both supine and tilt position in PCOS cases. 2) Positive correlation between the LF/HF ratio and the testosterone/ androstenedione ratio 3) Negative correlation between the LF/HF ratio and androstenedione
Kilit et al, 2017 [22]	Case- control	Turkey	English	Rotterdam	Normal	60	60	RMSSD, SDNN, mean-RR, HF, LF, HFnu, LFnu, LF/HF ratio	Age, BMI, SBP, DBP	No significant changes of HRV measures in PCOS cases.
Velusami et al, 2018 [24]	Cross-sectional	India	English	Rotterdam	Overweight	30	30	RMSSD, SDNN, Mean-RR, LFnu, HFnu, LF/HF, TP	Age, BMI, SBP, DBP, Baseline heart rate	1) Significant changes of HRV measures in PCOS cases. 2) Significant association of BMI with sympathovagal imbalance.
Ji et al, 2018 [25]	Cross-sectional	Korea	English	Rotterdam	Normal	35	32	RMSSD, SDNN, HF, LF, HFnu, LFnu, LF/HF ratio	Age, BMI, Baseline heart rate, SBP, DBP	Significant changes of HRV measures in PCOS cases.
Philbois et al, 2019 (1) [46]	Case- control	Brazil	English	Rotterdam (classic type)	Overweight	30	30	mean-RR, HF, LF, HFnu, LFnu, LF/HF ratio	Age, BMI, Baseline heart rate, SBP, DBP	No significant change in autonomic cardiovascular control in non-obese PCOS cases.
Philbois et al, 2019 (2) [46]	Case- control	Brazil	English	Rotterdam (classic type)	Obese	30	30	mean-RR, HF, LF, HFnu, LFnu, LF/HF ratio	Age, BMI, Baseline heart rate, SBP, DBP	Significant attenuated HRV values in obese-PCOS cases.
Mishra et al,2019 [42]	Case- control	India	English	Rotterdam	Normal	27	25	RMSSD, SDNN, NN50, LF, HF, LF/HF, TP	Age, BMI	1) Significant changes of HRV measures in PCOS cases. 2) Isotonic exercise challenge can be a useful tool in the assessment of autonomic fitness in PCOS patients.
Ribeiro et al, 2020 [43]	Case- control	Brazil	English	Rotterdam	Overweight	32	32	RMSSD, mean-RR, HF, LF, HFnu, LFnu, LF/HF ratio	Age, BMI	Altered cardiac autonomic activity in both supine and tilt position in PCOS cases.

*HF* High frequency, *HFnu* Normalized unit of high frequency, *LF* Low frequency, *LFnu* Normalized unit of low frequency, *LF/HF* ratio of low-frequency and high-frequency power, *RMSSD* the root mean square of successive R–R interval differences, *SDNN* Standard deviation of all NN intervals, *SDANN* Standard deviation of averages of NN intervals, *pNN50* percentage of NN50, *RMSSD* square root of the mean squared differences of successive NN intervals, *TP* Total power, *BMI* Body mass index, *DBP* Diastolic blood pressure, *SBP* Systolic blood pressure, *WHO* World health organization, *NIH* National institute of health

### Quality assessment

**Fig. 2:**
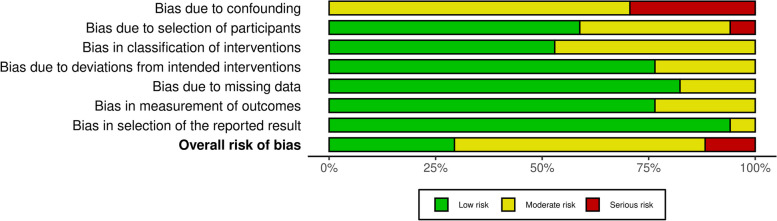
ROBINS 1 risk of bias summary

**Fig. 3:**
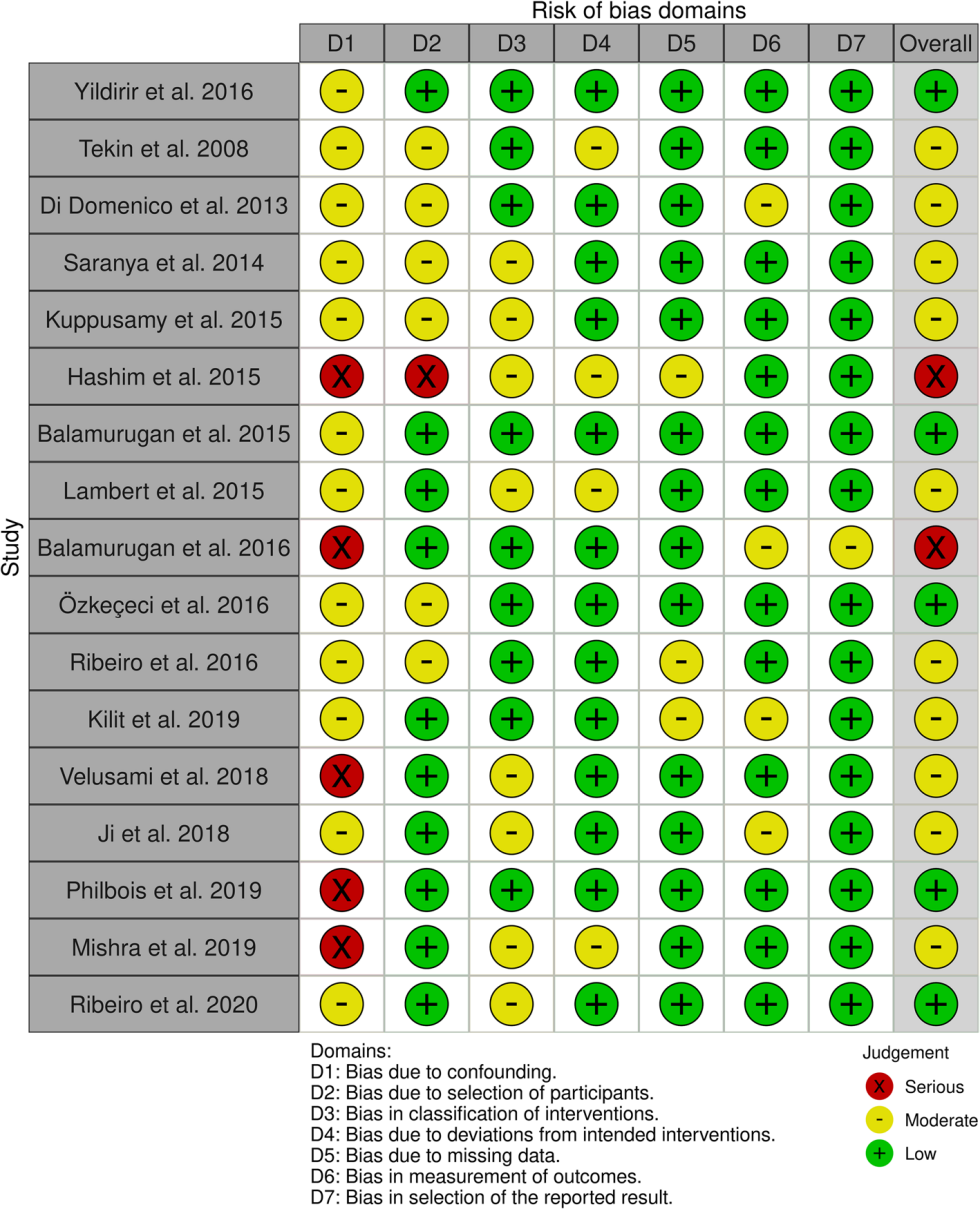
Traffic light plot risk of bias assessment (ROBINS-1) for each study

Generally, the risk of overall bias was estimated as moderate (see Fig. 2). Out of the 17 studies scrutinized, 5 exhibited a serious risk of confounding bias owing to the omission of crucial confounders, notably the blood sugar profile, IR measurement profile, and androgen profile. A single study was identified as having a notable risk of selection bias due to an imperfect match between the control and case groups. In summary, 2 studies were deemed to possess a substantial risk of bias, while 10 studies were categorized as having a moderate risk of bias across at least three domains. The remainder were deemed to have a low risk of bias (see Fig. 3). The certainty of evidence for outcomes, as assessed by GRADE framework, is delineated in Table 2. The meta-analysis indicates a moderate level of certainty in the majority of findings, primarily attributable to the inherent susceptibility to bias in observational studies, potential bias in the selection and diagnosis of PCOS cases, substantial heterogeneity, and reporting bias arising from the limited diversity in the countries of origin for the included studies. Furthermore, two outcomes exhibit a low level of certainty due to an insufficient number of studies included in the meta-analysis.

**Table 2:** GRADE evidence profile for HRV outcomes

**Quality assessment**								**Summary of findings**			**Certainty**
**Outcome**	**No of studies (Design)**	**Risk of bias**	**Limitation**	**Inconsistency**	**Indirectness**	**Imprecision**	**Other consideration**	**Number of patients**		**Std mean difference (95% CI)**	
								**PCOS**	**Control**		
**Frequency-domain measures**											
LF/HF ratio	5 (cross-sectional) 9 (case-control)	Moderate^1,2^	Low	High	Low	Low	Reporting bias^4^	440	425	0.670 [0.248, 1.091]	⊕ ⊕ ⊕◯Moderate
HFnu	5 (cross-sectional) 8 (case-control)	Moderate^1,2^	Low	High	Low	Low	Reporting bias^4^	413	400	-0.873 [-1.257, -0.489]	⊕ ⊕ ⊕◯Moderate
LFnu	5 (cross-sectional) 8 (case-control)	Moderate^1,2^	Low	High	Low	Low	Reporting bias^4^	413	400	0.840 [0.428, 1.251]	⊕ ⊕ ⊕◯Moderate
HF	2 (cross-sectional) 9 (case-control)	Moderate^1,2^	Low	High	Low	Low	Reporting bias^4^	352	343	-0.75 [-1.267, -0.239]	⊕ ⊕ ⊕◯Moderate
LF	2 (cross-sectional) 9 (case-control)	Moderate^1,2^	Low	High	Low	Low	Reporting bias^4^	352	343	-0.273 [-0.800, 0.254]	⊕ ⊕ ⊕◯Moderate
TP	3 (cross-sectional) 3 (case-control)	Moderate^2^	Moderate	High	Low	Moderate^3^	Reporting bias^4^	173	165	-1.997 [-3.306, -0.687]	⊕ ⊕◯◯Low
**Time domain measures**											
RMSSD	7 (cross-sectional) 7 (case-control)	Moderate^1,2^	Low	High	Low	Low	Reporting bias^4^	436	398	-0.274 [-0.616, 0.067]	⊕ ⊕ ⊕◯Moderate
Mean-RR	4 (cross-sectional) 7 (case-control)	Moderate^1,2^	Low	High	Low	Low	Reporting bias^4^	348	338	-0.174 [-0.816, 0.469]	⊕ ⊕ ⊕◯Moderate
PNN50	5 (cross-sectional) 8 (case-control)	Moderate^2^	Moderate	High	Low	Low	Reporting bias^4^	210	173	-1.245 [-2.07, -0.419]	⊕ ⊕ ⊕◯Moderate
NN50	4 (case-control)	Low	High	Low	Moderate	High^3^	None	117	111	-0.828 [-1.1, -0.556]	⊕ ⊕◯◯Low
SDNN	6 (cross-sectional) 6 (case-control)	Moderate^1,2^	Moderate	High	Low	Low	Reporting bias^4^	374	343	-0.763 [-1.289, -0.237]	⊕ ⊕ ⊕◯Moderate
SDANN	2(cross-sectional) 2 (case-control)	Low	High	Low	Moderate	High^3^	None	113	89	-0.507 [-0.793, -0.221]	⊕ ⊕◯◯Low

1. Three studies involved only classic phenotype of PCOS cases (selection bias)

2. The majority of the studies employed the Rotterdam criteria for diagnosing PCOS (detection bias)

3. A limited number of studies have addressed the HRV parameter

4. The predominant proportion of studies originates from India, Brazil, and Turkey

### Result of synthesis

The meta-analysis findings of HRV parameters, organized into two classifications of time-domain and frequency-domain measures, are detailed as follows:

### Time-domain measures

The meta-analysis revealed a significant difference in SDNN, SDANN, PNN50, and NN50 between PCOS cases and the control group, indicating an increased level of these measures in PCOS cases compared to the control group with SMD (CI95%) of -0.763 (95%CI [-1.289, -0.237], *p*=0.004), -0.507 (95%CI [-0.793, -0.221], *p*=0.000), -1.245 (95%CI [-2.07, -0.419], *p*=0.003) and -0.828 (95%CI [-1.1, -0.556], *p*=0.000), respectively. In terms of heterogeneity, SDNN (90.8%) and PNN50 (92.2%) displayed high levels, while SDANN (43.8%) and NN50 (40.9%) demonstrated a comparatively lower level. There was no significant difference in terms of RMSSD and mean-RR between PCOS cases and control group with SMD (CI95%) of -0.274 (95%CI [-0.616, 0.067], *p*=0.116) and -0.174 (95%CI [-0.816, 0.469], *p*=0.596), respectively. Table 3 provides a comprehensive overview of the detailed findings.

**Table 3:** Results of HRV measures

**Parameters**	**No. Studies**	**No. Cases**	**No. Controls**	**Effect Model**	**SMD (CI: 95%)**	***P* value**	**Heterogeneity**	
							**I2**	***P* value**
**Time-domain measures**								
**RMSSD**								
Overall	14	436	398	Random	-0.274 [-0.616, 0.067]	0.116	82.7%	0.000
**1. Grouped by BMI**								
Normal weight	5	169	166	Random	-0.416 [-0.953, 0.121]	0.129	56.1%	0.058
Overweight	4	128	124	Random	**-0.660 [-1.261, -0.060]**	**0.031**	78.5%	0.003
Obese	3	81	64	Random	0.585 [-0.137, 1.308]	0.112	91.4%	0.000
Not mentioned	2	58	44	Random	-0.355 [-1.222, 0.511]	0.421	83%	0.015
**2. Grouped by study design**								
Cross-sectional	7	225	187	Random	-0.451 [-0.940, 0.038]	0.071	78.1%	0.000
Case-control	7	211	211	Random	-0.094 [-0.587, 0.4]	0.710	86.2%	0.000
**Mean-RR**								
Overall	11	348	338	Random	-0.174 [-0.816, 0.469]	0.596	93.6%	0.000
**1. Grouped by BMI**								
Normal weight	3	114	114	Random	-0.219 [-1.577, 1.139]	0.752	76.2%	0.015
Overweight	5	155	150	Random	-0.419 [-1.475, 0.637]	0.437	92.1%	0.000
Obese	3	79	74	Random	0.309 [-1.079, 1.697]	0.663	97.8%	0.000
**2. Grouped by study design**								
Cross-sectional	4	126	115	Random	-0.641 [-1.711, 0.428]	0.24	91.5	0.000
Case-control	7	222	223	Random	0.097 [-0.716, 0.910]	0.815	94.3	0.000
**SDNN**								
Overall	12	374	343	Random	**-0.763 [-1.289, -0.237]**	**0.004**	90.8%	0.000
**1. Grouped by BMI**								
Normal weight	5	169	166	Random	**-0.98 [-1.875, -0.085]**	**0.032**	94.8%	0.000
Overweight	3	96	92	Random	-1.053 [-2.180, 0.074]	0.067	51.2%	0.129
Obese	2	51	41	Random	-0.082 [-1.48, 1.316]	0.909	93.2%	0.000
Not mentioned	2	58	48	Random	-0.565 [-1.954, 0.824]	0.426	79.4%	0.027
**2. Grouped by study design**								
Cross-sectional	6	195	164	Random	-0.643 [-1.416, 0.129]	0.103	83.5%	0.000
Case-control	6	179	179	Random	**-0.910 [-1.703, -0.117]**	**0.025**	94.2%	0.000
**SDANN**								
Overall	4	113	89	Fixed	**-0.507 [-0.793, -0.221]**	**0.000**	43.81%	0.149
**PNN50**								
Overall	7	210	173	Random	**-1.245 [-2.07, -0.419]**	**0.003**	92.2%	0.000
**1. Grouped by BMI**								
Normal weight	1	24	24		-0.867 [-1.459, -0.275]	-	-	-
Overweight	2	66	62	Fixed	**-2.688 [-3.166, -2.209]**	**0.000**	0%	0.67
Obese	2	62	43	Random	-0.653 [-1.803, 0.496]	0.265	95.1%	0.000
Not mentioned	2	58	44	Fixed	**-0.597 [-1.00, -0.194]**	**0.004**	0%	0.384
**2. Grouped by study design**								
Cross-sectional	5	160	125	Random	**-9.71 [-18.00, -1.42]**	**0.002**	96.4%	0.000
Case-control	2	50	48	Fixed	**-0.822 [-1.235, -0.410]**	**0.000**	0%	0.835
**NN50**								
Overall	4	117	111	Fixed	**-0.828 [-1.1, -0.556]**	**0.000**	40.94%	0.166
**Frequency-domain measures**								
**LF/HF ratio**								
Overall	14	440	425	Random	**0.670 [0.248, 1.091]**	**0.002**	88.5%	0.000
**1. Grouped by BMI**								
Normal weight	5	176	171	Random	0.538 [-0.185, 1.262]	0.145	89%	0.000
Overweight	6	185	180	Random	**1.006 [0.343, 1.669]**	**0.003**	83.3%	0.000
Obese	3	79	74	Random	0.209 [-0.739, 1.157]	0.665	94.1%	0.000
**2. Grouped by study design**								
Cross-sectional	5	161	147	Fixed	**0.638 [0.407, 0.868]**	**0.000**	29.8%	0.223
Case-control	9	279	278	Random	**0.689 [0.138, 1.241]**	**0.014**	92.5%	0.000
**HFnu**								
Overall	13	413	400	Random	**-0.873 [-1.257, -0.489]**	**0.000**	85.1%	0.000
**1. Grouped by BMI**								
Normal weight	4	149	146	Random	**-0.936 [-1.676, -0.196]**	**0.013**	81.8%	0.001
Overweight	6	185	180	Random	**-0.992 [-1.601, -0.384]**	**0.001**	84.7%	0.000
Obese	3	79	74	Random	-0.549 [-1.419, 0.322]	0.217	92.7%	0.000
**2. Grouped by study design**								
Cross-sectional	5	161	147	Fixed	**-0.702 [-0.934, -0.470]**	**0.000**	47.7%	0.105
Case-control	8	252	253	Random	**-0.986 [-1.499, -0.473]**	**0.000**	90.2%	0.000
**LFnu**								
Overall	13	413	400	Random	**0.840 [0.428, 1.251]**	**0.000**	86.9%	0.000
**1. Grouped by BMI**								
Normal weight	4	149	146	Random	**0.961 [0.176, 1.746]**	**0.016**	81.7%	0.001
Overweight	6	185	180	Random	**0.991 [0.355, 1.308]**	**0.003**	83.8%	0.000
Obese	3	79	74	Random	0.365 [-0.559, 1.288]	0.439	94.8%	0.000
**2. Grouped by study design**								
Cross-sectional	5	161	147	Fixed	**0.776 [0.543, 1.010]**	**0.000**	33.8%	0.196
Case-control	8	252	253	Random	**0.885 [0.332, 1.437]**	**0.002**	91.8%	0.000
**HF**								
Overall	11	352	343	Random	**-0.75 [-1.267, -0.239]**	**0.004**	90.3%	0.000
**1. Grouped by BMI**								
Normal weight	5	176	171	Random	-0316 [-1.181, 0.548]	0.473	58.23%	0.048
Overweight	4	120	118	Random	**-1.352 [-2.348, -0.357]**	**0.008**	96.5%	0.000
Obese	1	30	30		-0.846 [-2.786, 1.094]	-	-	-
Not mentioned	1	26	24		-0.847 [-1.426, -0.268]	-	-	-
**2. Grouped by study design**								
Cross-sectional	2	66	62	Random	**-2.535 [-3.829, -1.241]**	**0.000**	98.7%	0.000
Case-control	9	287	281	Random	-0.409 [-0.981, 0.163]	0.161	55%	0.023
**LF**								
Overall	11	352	343	Random	-0.273 [-0.800, 0.254]	0.310	91.12%	0.000
**1. Grouped by BMI**								
Normal weight	5	176	171	Fixed	0.112 [-0.099, 0.323]	0.297	0%	0.650
Overweight	4	120	118	Random	0.007 [-0.634, 0.647]	0.984	92.9%	0.000
Obese	1	30	30		-0.590 [-1.885, 0.706]	-	-	-
Not mentioned	1	26	24		-3.043 [-3.786, -2.299]	-	-	-
**2. Grouped by study design**								
Cross-sectional	2	66	62	Random	-0.575 [-1.849, 0.699]	0.3766	94.1%	0.000
Case-control	9	287	281	Random	-0.207 [-0.810, 0.396]	0.501	91.2%	0.000
TP								
Overall	6	173	165	Random	**-1.997 [-3.306, -0.687]**	**0.003**	95%	0.000
**1. Grouped by BMI**								
Normal weight	2	53	49	Fixed	**-0.445 [-0.843, -0.046]**	**0.029**	34.2%	0.217
Overweight	3	96	923	Random	**-3.473 [-5.616, -1.329]**	**0.001**	97.6%	0.000
Not mentioned	1	26	24		-0.879 [-1.460, 0.298]	-	-	-
**2. Grouped by study design**								
Cross-sectional	3	96	92	Random	**-3.444 [-5.232, -1.656]**	**0.000**	97.6%	0.000
Case-control	3	77	73	Fixed	**-0.583 [-0.912, -0.255]**	**0.001**	32.8%	0.225

### Frequency-domain measures

The PCOS cases showed a significant difference in all frequency-domain measures, except for LF, when compared to the control group. A significant rise in the LF/HF ratio and LFnu was observed in PCOS cases in comparison to the control group, showed by SMDs (CI95%) of 0.670 (95%CI [0.248, 1.091], p=0.002) and 0.840 (95%CI [0.428, 1.251], *p*=0.000), respectively. Moreover, PCOS cases exhibited a notable reduction in HF, HFnu, and TP in comparison to the control group with SMDs (CI95%) of -0.75 (95%CI [-1.267, -0.239], *p*=0.004), -0.873 (95%CI [-1.257, -0.489], *p*=0.000) and -1.997 (95%CI [-3.306, -0.687], *p*=0.003), respectively. A high degree of heterogeneity exceeding 80% was observed across all measures. No significant distinction was found in the LF band when comparing the two groups of individuals with PCOS and the control group with a SMD (CI95%) of -0.273 (95% CI [-0.800, 0.254], *p*=0.310).

Table 3 provides a comprehensive overview of the detailed findings.

## Subgroup analysis and investigations of heterogeneity

### Grouped by BMI

#### Time-domain measures

A notable decline in RMSSD and PNN50 among overweight PCOS cases compared to their respective control group with SMDs of -0.660 (95%CI [-1.261, -0.060], p= 0.031) and -2.688 (95%CI [-3.166, -2.209], p=0.000), respectively. RMSSD showed a high heterogeneity (78.5%), whereas, PNN50 revealed 0% heterogeneity. Normal-weighted PCOS cases also demonstrated lower SDNN with a SMD of -0.98 (95%CI [-1.875, -0.085], p=0.032) in comparison with the control group. SDNN displayed a significant level of high heterogeneity (94.8%). There were no statistically significant findings in the obese category for any of these HRV measures (see Table 3).

#### Frequency-domain measures

The meta-analysis disclosed significant differences in the LF/HF ratio, HFnu, LFnu, HF, and TP between overweight individuals with PCOS and their corresponding control groups. Overweight PCOS cases exhibited a significant increase in both LF/HF ratio and LFnu compared to the control group with SMDs (CI95%) of 1.006 (95%CI [0.343, 1.669], p=0.003) and 0.991 (95%CI [0.355, 1.308], p=0.003), respectively. Additionally, HFnu, HF, and TP were significantly reduced in overweight PCOS cases in comparison to the controls with SMDs (CI95%) of -0.992 (95%CI [-1.601, -0.384], p=0.001), -1.352 (95%CI [-2.348, -0.357], p=0.008) and -3.473 (95%CI [-5.616, -1.329], p=0.001), respectively. LFnu, HFnu and TP were also revealed to be significantly different in normal-weight PCOS cases compared to control group with SMDs (CI95%) of 0.961 (95%CI [0.176, 1.746], p=0.016) and -0.936 (95%CI [-1.676, -0.196], p=0.013) and-0.445 (95%CI [-0.843, -0.046], p=.029), respectively. These findings exhibited a notable degree of high heterogeneity. No statistically significant findings were observed in the obese category for any of these HRV measures (see Table 3).

#### Grouped by study design

The classification of articles into two groups, based on their study designs-cross-sectional and case-control-did not result in significant alterations in the overall discoveries. Both types of designs presented significant findings concerning the HRV measures mentioned. Regarding heterogeneity, in cross-sectional category, a significant decrease was observed in LF/HF ratio (29.8%), LFnu (3.8%) and HFnu (47.7%). In case-control category, PNN50, HF and TP showed a lower heterogeneity (0%, 5%, 32.8%, respectively) comparing to the overall analysis (see Table 3).

Forest plots of meta-analysis are provided as supplement (**S4-document**).

#### Anthropometric features

There was no significant age difference between PCOS cases and control group, indicated by a SMD (CI95%) of -0.301 (95%CI [-0.737, 0.134], p=0.175). PCOS cases showed a significantly higher BMI compared to the control group demonstrating a SMD (CI95%) of 0.864 (95%CI [0.344, 1.384], p=0.001). In terms of blood pressure, individuals with PCOS exhibited elevated levels of both SBP and DBP when compared to control groups as indicated by SMDs (CI95%) of 0.580 (95%CI [0.152, 1.009], p=0.008) for SBP and 0.754 (95%CI [0.339, 1.170], p=0.000) for DBP, respectively. No significant difference was observed in baseline heart rate between PCOS cases and control group with a SMD (CI95%) of 0.089 (95%CI [-0.495, 0.672], p=0.766). Significant heterogeneity was noted across all anthropometric characteristics (see Table 4).

**Table 4:** Anthropometric features

**Parameters**	**No. Studies**	**No. Cases**	**No. Controls**	**Effect Model**	**SMD (CI: 95%)**	***P* value**	**Heterogeneity**	
							**I2**	***P* value**
Age	14	437	423	Random	-0.301 [-0.737, 0.134]	0.175	89.5%	0.000
BMI	17	519	494	Random	**0.864 [0.344, 1.384]**	**0.001**	93.2%	0.000
Baseline Heart rate	12	338	334	Random	0.089 [-0.495, 0.672]	0.766	92.3%	0.000
SBP	16	492	457	Random	**0.580 [0.152, 1.009]**	**0.008**	89.9%	0.000
DBP	16	492	457	Random	**0.754 [0.339, 1.170]**	**0.000**	89.1%	0.000

*BMI* Body mass index, *SBP* Systolic blood pressure, *DBP* Diastolic blood pressure

#### *Meta*-regression

**Fig. 4:**
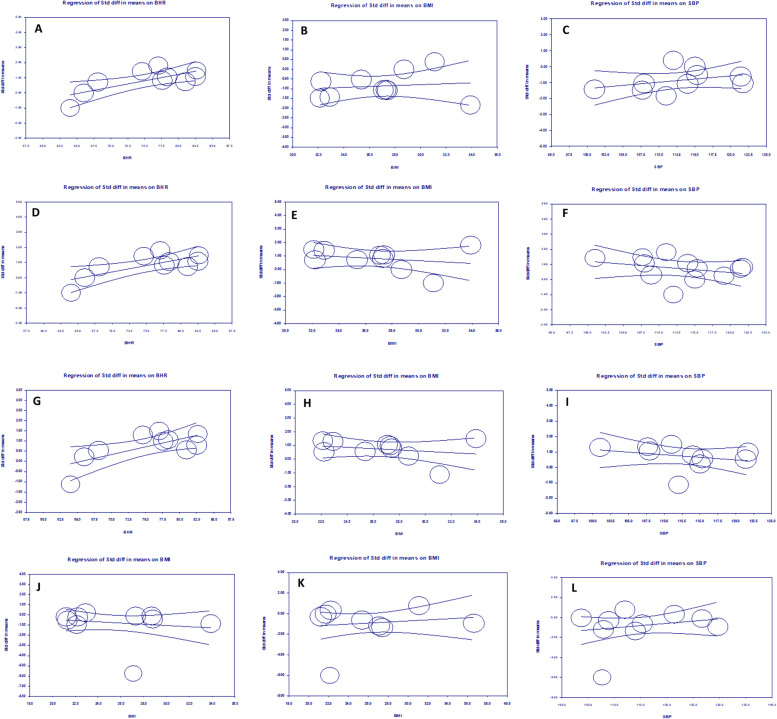
Meta-regression plots for HFnu-BHR, HFnu-BMI, HFnu-SBP, LFnu-BHR, LFnu-BMI, LFnu-SBP, LF/HF-BHR, LF/HF-BMI, LF/HF-SBP, HF-BMI, SDNN-BMI, and SDNN-SBP represented from A-L

The results of univariate meta-regression, revealed a significant positive association of BHR with LF/HF ratio and LFnu. On average, one unit increase in the BHR, was associated with increase in the SMDs of LF/HF and LFnu with effect sizes of 0.0754 (95%CI [0.023, 0.126], *p*=0.004) and 0.0845 (95CI [0.0317, 0.1372], p=0.001), respectively. This moderator accounted for nearly half amount of the heterogeneity in both LF/HF and LFnu (R2 ≈0.5). Moreover, a significant reverse correlation was observed between HFnu and BHR with an effect size of -0.069 (95%CI [-0.117, -0.0217], *p*=0.004). BHR could explain almost %50 of heterogeneity in HFnu (R^2^=0.49). No significant correlations were seen with BMI and SBP as potential moderators (see Table 5 and Fig. 4).

**Table 5:** Results of Meta-regression test

Moderator	Coefficient	SE	Z value	*P* value	95% CI	R^2^
**LF/HF**						
BHR	0.0754	0.0263	2.87	**0.004**	0.023 to 0.126	0.44
BMI	-0.0498	0.0605	-0.82	0.411	-0.168 to 0.0689	0.00
SBP	-0.0338	0.0354	-0.96	0.339	-0.103 to 0.0355	0.00
**HFnu**						
BHR	-0.069	0.024	-2.85	**0.004**	-0.117 to -0.0217	0.49
BMI	0.0236	0.059	0.40	0.688	-0.092 to 0.139	0.00
SBP	0.039	0.033	1.19	0.234	-0.0257 to 0.104	0.04
**LFnu**						
BHR	0.0845	0.0269	3.14	**0.001**	0.0317 to 0.1372	0.51
BMI	-0.0504	0.0655	-0.77	0.441	-0.178 to 0.0780	0.00
SBP	-0.037	0.0337	-1.11	0.267	-0.103 to 0.0287	0.00
**HF**						
BMI	-0.0604	0.0735	-0.82	0.411	-0.204 to 0.0837	0.00
**SDNN**						
BMI	0.0516	0.0773	0.67	0.504	-0.0999 to 0.203	0.00
SBP	0.0442	0.0412	1.07	0.283	-0.0365 to 0.124	0.00

#### Publication *bias*

**Fig. 5:**
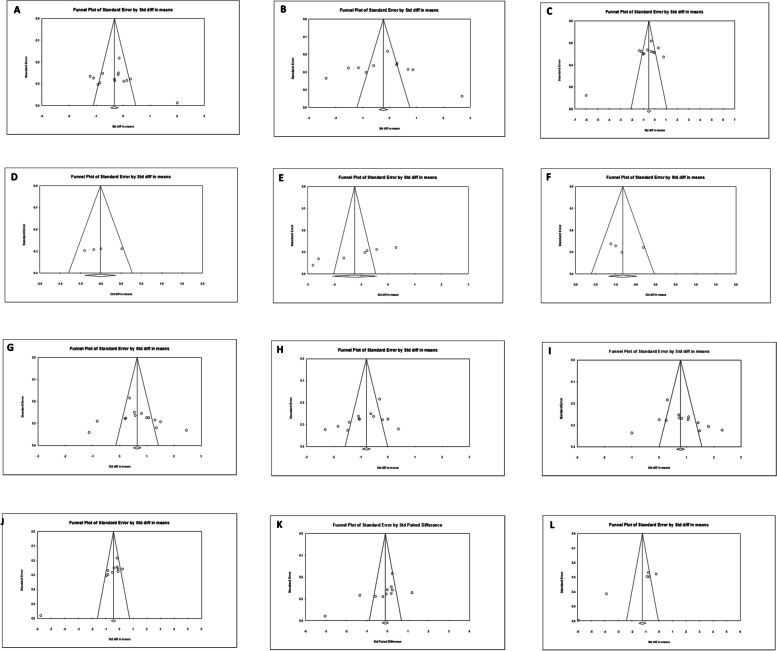
Funnel plots for RMSSD (**A**), mean-RR (**B**), SDNN (**C**), SDANN (**D**), PNN50 (**E**), NN50 (**F**), LF/HF ratio (**G**), HFnu (**H**), LFnu (**I**), HF (**J**), LF (**K**), TP (**L**), respectively

**Fig. 6:**
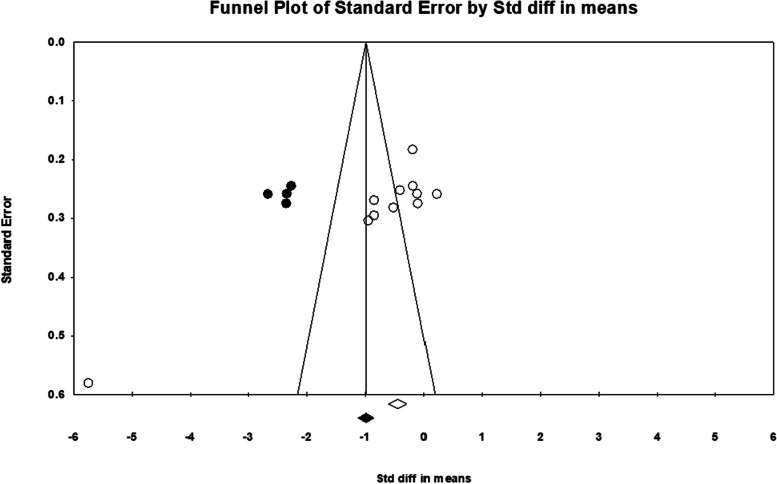
Funnel plot with the trim and fill method for meta-analysis regarding HF band. Open circles indicate observed studies, whereas filled circles indicate imputed studies. Four studies added on the left side, yielding a summary effect size of -1.235, with 95% CI = (-1.828, -0.624)

**Fig. 7:**
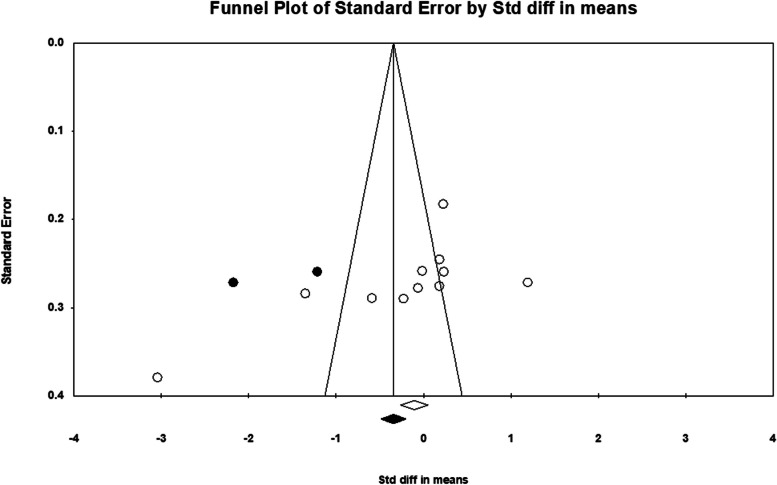
Funnel plot with the trim and fill method for meta-analysis regarding LF band. Open circles indicate observed studies, whereas filled circles indicate imputed studies. Two studies added on the left side, yielding a summary effect size of -0.497, with 95% CI = (-1.053, 0.058)

Funnel plot analysis (see Fig. 5) and Begg’s and Egger’s tests (see Table 6) were used to assess the publication bias. An evident publication bias was noted in the examination of the LF and HF bands. The adjustment from the Duval and Tweedie’s trim and fill method suggested that four studies needed to be imputed on the left side of the scatter plot for HF band analysis (see Fig. 6). Following this correction, the summary effect size was -1.235, with 95% CI = (-1.828, -0.624). In the case of LF band analysis (see Fig. 7), two studies required imputation on the left side of the scatter plot. This correction resulted in a summary effect size of -0.497, with 95% CI = (-1.053, 0.058).

**Table 6:** Begg’s test and Egger’s test for publication bias

Analysis value	Study (n)	Egger’ test		Beggs’ test			
		*i*	*p*	*k*	*t*	*z*	*p*
RMSSD	14	*3.51*	*0.40*	*3*	*0.021*	*0.109*	*0.91*
Mean-RR	11	*1.56*	*0.809*	*-1.00*	*0.000*	*0.000*	*1.000*
SDNN	12	*-7.98*	*0.043*	*-6.00*	*-0.075*	*0.342*	*0.731*
LF/HF ratio	14	*2.774*	*0.602*	*17.00*	*0.175*	*0.875*	*0.381*
HFnu	13	*-7.092*	*0.121*	*-22.00*	*-0.269*	*1.28*	*0.200*
LFnu	13	*5.192*	*0.294*	*20.00*	*0.243*	*1.159*	*0.246*
HF	11	*-11.54*	0.001*	*-33.00*	*-0.581*	*2.491*	0.012*
LF	11	*-11.39*	0.049*	*-35.00*	*-0.618*	*2.646*	0.008*

*i* intercept*, p p-*value*, k* Kendall’s Score*, t* tau*, z z-*value

#### Sensitivity analysis

**Fig. 8:**
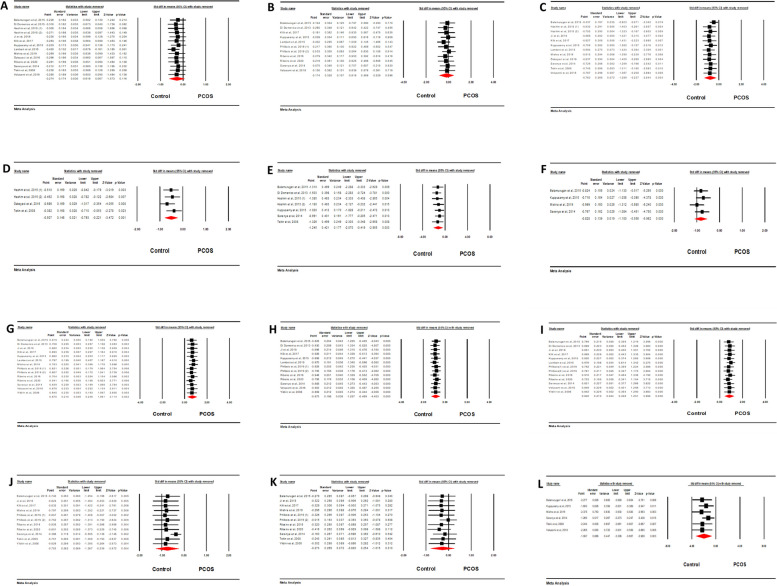
Forest plot of sensitivity analysis for RMSSD (**A**), mean-RR (**B**), SDNN (**C**), SDANN (**D**), PNN50 (**E**), NN50 (**F**), LF/HF ratio (**G**), HFnu (**H**), LFnu (**I**), HF (**J**), LF (**K**), TP (**L**), respectively

Upon visual examination of the forest plots (see Fig. 8), no apparent outliers were observed in any of the outcomes. This suggests that the likelihood of a single study significantly influencing or biasing the mean difference in either direction is low.

## Discussion

HRV is a statistical parameter and noninvasive approach that quantifies the cyclic variation in heart rate between consecutive beats. This measure is indicative of the individual's cardiac autonomic activity and is extensively utilized to assess the risk associated with various cardiac and noncardiac disorders [47]. Women with PCOS are identified by chronic anovulation, which occurs along with excess androgen, IR, and changes in gonadotropin secretion. In addition to the heightened risk of reproductive abnormalities associated with PCOS, most women with this condition are also at a high risk of developing cardiovascular disease [48].

In this meta-analysis, distinct variations in HRV measures were observed among PCOS cases, both in the time domain (SDNN, SDANN, PNN50, NN50) and frequency-domain (LF/HF, LFnu, HFnu, HF, TP), when compared to non-PCOS controls.

In a comprehensive review and meta-analysis conducted by Gui et al., the analysis of data from 7 studies examining HRV revealed altered measures in PCOS cases. Regarding time domain measures, both SDNN and pNN50 were significantly lower in PCOS cases compared to controls. The meta-analysis of SDNN involved four studies, while pNN50 comprised only five studies. Importantly, other time domain indices, including mean-RR, NN50, and SDANN, were not subjected to meta-analysis. Also, there were no significant disparities found in the frequency domain measures, encompassing LF/HF ratio, LFnu, HFnu, LF, and HF bands, between PCOS cases and controls. It is noteworthy that only TP displayed a noticeable reduction in PCOS cases. It is crucial to highlight that the TP findings were based on a mere two studies that were incorporated in the meta-analysis.[9].

Moreover, in this review, a subgroup meta-analysis was carried out to explore the impact of BMI on HRV measures. The results revealed that overweight individuals with PCOS displayed significant deviations in both time-domain metrics (RMSSD, mean-RR, SDNN, PNN50) and frequency-domain measures (LF/HF ratio, HFnu, LFnu, HF, TP) compared to the non-PCOS controls. In contrast, obese PCOS cases did not manifest any notable changes in HRV measures when compared to their corresponding control groups. Additionally, PCOS cases with normal weight exhibited significant variations in certain HRV measures (SDNN, HFnu, LFnu, TP) in comparison to non-PCOS controls.

In this meta-analysis, 17 studies were considered, but only four specifically examined cases of PCOS in individuals categorized as obese. The findings from this review regarding obese cases are consistent with just one of these studies, which reported no significant changes in HRV when comparing PCOS cases to control groups.

Conversely, Hashim et al., Philbois et al., and Di Domenico et al. reported significant variations in HRV among PCOS cases compared to their respective control groups[38, 39, 46]. Specifically, Hashim et al. documented a significant reduction in SDNN and PNN50 in obese PCOS cases in comparison to obese non-PCOS controls[39]. In the investigation by Di Domenico et al., obese PCOS cases exhibited modified HRV measures, including LFnu, HFnu, LF/HF ratio, mean-RR, RMSSD, and PNN50, both at rest and during mental stress when compared to an overweight control group [38]. Philbois et al. noted a significant decrease in HFnu and an increase in the LF band in obese PCOS cases compared to normal-weight controls[46]. In the study conducted by Lambert et al., obese PCOS cases demonstrated no significant changes in either time-domain or frequency-domain HRV when compared to obese control cases [20].

There is limited research available in the literature regarding the relationship between obesity and PCOS. The existing studies present conflicting findings, with some suggesting that this association may have a detrimental impact on HRV[37, 49], although others report that there is no association between weight gain and PCOS [20, 38].

Numerous studies have provided evidence of a negative correlation between weight gain and changes in HRV parameters[50–52]. Additionally, vagal modulation exhibited an inverse relationship with body fat percentage, elevated body mass, and waist circumference [53–56]. Overweight individuals displayed a sympathovagal imbalance due to increased sympathetic activity associated with visceral fat [57]. Fat percentage and waist-to-hip ratio were found to have a negative correlation with both RMSSD and LF band [58]. A potential association between obesity mechanisms and a decreased sympathetic system response in the postsynaptic region has been identified, as evidenced by the elevated concentration of noradrenaline in the presynaptic cleft [59, 60]. However, HRV in individuals with overweight and obesity can be influenced by various determinants, including co-morbidities, dietary habits, physical activity levels, emotional stress, and genetic factors[11, 61–63].

The variations in PCOS phenotypes, the presence and severity of insulin resistance (IR), the distribution of adipose tissue, the levels of physical activity, and the relatively small sample size may explain the discrepancy in the significance of HRV findings observed between overweight and obese individuals with PCOS compared to the control group in this systematic review and meta-analysis.

The impact of IR and hyperinsulinemia on elevating sympathetic outflow through central brain receptors is well-established [64]. Saito et al. conducted a study revealing that reduced HRV was linked to IR and lower insulin sensitivity [65]. Furthermore, a decline in the insulin sensitivity index was found to be connected to parasympathetic dysfunction, primarily observed in overweight individuals [65, 66].

The findings of Kuppusamy et al. demonstrated a significant connection between the LF/HF ratio and HOMA-IR. Additionally, their study revealed that HOMA-IR independently influenced the LF/HF ratio, suggesting that IR may contribute to the sympathovagal imbalance observed in PCOS [36]. This meta-analysis unveiled that the PCOS cases included in the review exhibited elevated levels of FBS and an increased HOMA-IR in comparison to the control group. Nevertheless, within the overall studies included in the review, there was a restricted number of studies that reported these parameters. Additionally, their study demonstrated a significant positive correlation between the LF/HF ratio and BMI. However, it is worth mentioning that BMI did not make an independent contribution to the LF/HF ratio. The researchers hypothesized that obesity does not have a significant influence on the development of sympathovagal imbalance in PCOS [36].

The established understanding of how sympathetic activity regulates blood pressure, surpassing parasympathetic influence, underlies the initiation and maintenance of blood pressure, as evidenced by various studies [67, 68]. In the previous review, there were no notable distinctions in SBP or DBP between individuals with PCOS and the control group. Nevertheless, our results revealed a significant elevation in both SBP and DBP among PCOS patients when compared to controls. This elevation may be attributed to heightened sympathetic activity observed in these individuals as suggested by the meta-analysis results.

Cardiac autonomic neuropathy (CAN) is widely recognized as a complication of diabetes mellitus. However, emerging evidence suggests that CAN can also be present in individuals with obesity, pre-diabetes, and metabolic syndrome (MetS) even before the onset of diabetes mellitus [69]. CAN leads to a disruption in the functioning of the sympathetic and parasympathetic nerve fibers that innervate the heart and blood vessels [70]. There is a significant correlation between elevated BMI and a heightened susceptibility to CAN [71]. A recent investigation conducted on individuals with regular glucose tolerance has demonstrated that an elevated waist-to-hip ratio, which signifies the presence of visceral adiposity, is connected to compromised control of cardiac autonomic function by both the parasympathetic and sympathetic nervous systems [72]. The Mets encompasses various clinical characteristics of PCOS, including insulin resistance, obesity, dyslipidemia, and hyperandrogenism. It is worth mentioning that Mets affects 43% of adult women and nearly one-third of adolescent teenagers who have been diagnosed with PCOS [73].

The known associations between MetS and CAN suggest that a considerable proportion of PCOS patients may play a role in the increasing prevalence of CAN [74]. In a research study that examined the heart rate recovery following a maximal cardiopulmonary exercise test in young overweight women with PCOS, it was observed that PCOS patients exhibited a significant decrease in heart rate recovery compared to healthy individuals [75]. This decline in heart rate recovery is indicative of autonomic dysfunction and is inversely correlated with BMI in overweight PCOS patients [75].

It is important to note that, in the context of this review, the term "HRV" is expounded upon, specifically in relation to exercise-induced or stress-induced HRV. The studies analyzed in this review specifically evaluated HRV through the application of exercise or stress as stimuli. The particular emphasis on these forms of HRV underscores the dynamic nature of autonomic nervous system regulation in response to physical exertion or stressors [76, 77]. By specifically focusing on exercise and stress-induced HRV, the objective of this review is to capture the intricate complexities of autonomic modulation within the PCOS population.

Furthermore, apart from exercise-induced HRV, the significance of visit-to-visit HRV should not be overlooked, as it is closely linked to the risk of experiencing adverse cardiovascular outcomes [78]. In contrast to the acute modulation of HRV observed during exercise or stress, visit-to-visit HRV explores the long-term trends and variations in heart rate patterns across multiple occasions [78, 79]. This approach provides valuable insights into the sustained effects of exercise interventions on the regulation of the autonomic nervous system in individuals with PCOS. Both visit-to-visit HRV and exercise-induced HRV are relevant in the context of CAN associated with metabolic syndrome or diabetes. Visit-to-visit HRV can provide insights into the chronic impact of these conditions on autonomic function over time, while exercise-induced HRV reflects the dynamic response of the autonomic nervous system to physical activity [80–82].

Understanding the distinctions between these two dimensions of HRV in the context of PCOS is crucial for comprehensively evaluating the impact of stressors on autonomic function and cardiovascular health in this population. The interplay between acute stress/exercise-induced HRV and the longer-term visit-to-visit patterns sheds light on the dynamic nature of autonomic modulation and its potential implications for managing cardiovascular risk factors associated with PCOS.

Overall, this study provided evidence of the association between PCOS and altered cardiac autonomic function. Identifying low HRV as an early marker of autonomic dysfunction suggests the potential for early interventions to prevent or manage cardiovascular complications in PCOS patients and the results may contribute to identifying subgroups within the PCOS population who are at higher cardiovascular risk based on their HRV profiles

### Limitations

The present meta-analysis possesses several limitations that necessitate addressing. Firstly, the number of studies assessing HRV in individuals with PCOS is notably limited. Moreover, the existing studies primarily focused on exercise-induced or stress-induced HRV, rather than visit-to-visit HRV. The majority of the enrolled participants in these studies were either of normal weight or overweight with PCOS, and only a few studies included assessments of HRV in obese cases. Furthermore, a significant proportion of the studies omitted reporting crucial HRV indices, including TP, RMSSD, SDNN, and PNN50. These indices are essential indicators for understanding HRV. Furthermore, the meta-analysis findings revealed substantial heterogeneity in most outcomes, and subgroup analysis was unable to fully elucidate the reasons behind this high heterogeneity. Another significant limitation is that not all studies classified their included PCOS cases into subgroups based on phenotypes and hormonal patterns, which may have contributed to the high heterogeneity in data and analysis. The lack of adequate information regarding participants' insulin resistance status, androgen profile, and lean body weight precluded subgroup analysis based on these factors to assess their impact on heterogeneity and overall results. One significant limitation of this study is the exclusive reliance on observational methodology in the design of the included articles. The absence of a structured follow-up of patients introduces a constraint on the depth of insight into long-term outcomes and may limit the establishment of causal relationships between variables. The inherent nature of observational studies poses challenges in controlling for confounding factors and establishing a cause-and-effect relationship. The assessment of the risk of bias in the included studies indicated that the majority exhibited a low to moderate risk of bias. Furthermore, employing the GRADE approach to determine the certainty of evidence for outcomes, a predominant number of studies achieved a moderate certainty score. Despite the significant findings, it is important to highlight the need for comprehensive studies with larger sample sizes and different subgroups with varying PCOS phenotypes to confirm and validate the results presented here.

### Implications for practice

Identifying low HRV as an early marker of autonomic dysfunction suggests the potential for early interventions to prevent or manage cardiovascular complications in PCOS patients. Furthermore, these findings may contribute to the identification of subgroups within the PCOS population, particularly overweight patients, who face a higher risk of cardiovascular problems based on their HRV profiles, allowing for tailored interventions. Also, irrespective of weight, a holistic approach to managing PCOS beyond weight management is essential. This may involve tailored interventions targeting factors influencing autonomic function, such as hormonal regulation, stress management, and lifestyle modifications.

### Implications for research

In order to offer precise recommendations, it is essential to conduct meticulously designed studies with large sample sizes. These studies should explore the impact of distinct PCOS phenotypes, insulin resistance, hyperandrogenism, adipose tissue distribution, and levels of physical activity on both exercise-induced-HRV and visit-to-visit HRV. Valuable insights into the generalizability of our findings could be gained through comparative analyses across diverse populations and ethnicities. Additionally, longitudinal studies could help elucidate the dynamic nature of HRV in PCOS, considering the potential impact of disease progression, lifestyle factors, and therapeutic interventions. Ultimately, this research emphasizes the importance of thorough examinations into the complex relationships among PCOS, body weight, and autonomic nervous system function. Addressing these research gaps will not only deepen our understanding of the physiological implications of PCOS but also pave the way for targeted interventions and personalized approaches in the management of PCOS-related cardiovascular health issues.

### Implication for public policy

These findings can contribute to public awareness campaigns aimed at educating the general population, healthcare professionals, and policymakers about the association between PCOS and cardiac autonomic dysfunction and highlighting the importance of early detection, screening, and appropriate management strategies for these patients. National and international health organizations develop clinical practice guidelines to standardize care and inform healthcare professionals about evidence-based practices. These findings can be considered in the development or revision of these guidelines, leading to recommendations for incorporating HRV assessments into the evaluation and management of PCOS patients. Policymakers and funding agencies rely on scientific evidence to allocate resources for research and healthcare initiatives.

### Future directions

Further research is needed to elucidate the underlying mechanisms linking PCOS and altered cardiac autonomic function. Exploring the hormonal, metabolic, and inflammatory factors that mediate the association can provide a deeper understanding of the pathophysiology and potential therapeutic targets. Additionally, conducting longitudinal studies can help establish the prognostic value of HRV in PCOS patients regarding subsequent cardiovascular events and their possible mortality.

## Conclusion

This meta-analysis emphasizes a significant link between overweight and normal-weighted PCOS cases and a decrease in HRV when compared to non-PCOS controls. It is worth noting that PCOS cases with obesity did not exhibit any significant changes in HRV. Nevertheless, the comprehensive analysis consistently reveals a tendency towards reduced HRV in PCOS cases, irrespective of their weight classification.

### Supplementary Information

Additional file 1: S1-document. PRISMA 2020 Checklist for systematic reviews and meta-analyses.

Additional file 2: S2-document. Table of search strategy conducted in online databases.

Additional file 3: S3-document. Table of excluded studies during final screening.

Additional file 4: S4-document. Forest plots of meta-analysis.

Additional file 5: High resolution funnel plots.

Additional file 6: High resolution meta-regression plots.

Additional file 7: High resolution sensitivity analysis forest plots.

## Data Availability

The datasets used or analyzed during the present study are available from the corresponding author on reasonable request and as the supplementary material appendix.

## Abbreviations

PCOS: Polycystic Ovary Syndrome
HRV: Heart Rate Variability
HF: High Frequency
HFnu: Normalized unit of High Frequency
LF: Low frequency
LFnu: Normalized unit of Low Frequency
LF/HF: ratio of low-frequency and high-frequency power
RMSSD: the root mean square of successive R–R interval differences
SDNN: Standard deviation of all NN intervals
SDANN: Standard Deviation of Averages of NN Intervals
PNN50: Percentage of NN50
RMSSD: Square Root of the Mean Squared Differences of successive NN intervals
TP: Total Power
