# Supplementary material for: Exploring heart rate variability in polycystic ovary syndrome: implications for cardiovascular health: a systematic review and meta-analysis
Source: Syst Rev. 2024 Jul 24;13:194. doi: 10.1186/s13643-024-02617-x (PMC11271026; doi:10.1186/s13643-024-02617-x)
Supplement: Supplementary file 2 — Additional file 2: S2-document. Table of search strategy conducted in online databases. [file 13643_2024_2617_MOESM2_ESM.docx]

**Table of Search Strategy**

| **Databases** | **Search terms** |
| --- | --- |
| **Web of Science** | #1 ALL=("heart rate variability" OR "cycle length variability" OR "r--r variability" OR "hrv" OR "heart rate recovery")  #2 TS=(parasympath* OR sympath* OR sympathovagal OR vagal OR vagus OR "autonom* nerv*" OR "ans" OR "pns" OR "sns")  #3 TS=("polycystic ovar* syndrome" OR "polycystic ovar* disease" OR "stein leventhal syndrome" OR "pcos" OR "sclerocystic ovar*" )  #4 #1 OR #2  #5 #4 AND #3 |
| **PubMed** | #1 "Autonomic Nervous System"[Mesh] OR "Vagus Nerve"[Mesh] OR "Parasympathetic Nervous System"[Mesh] OR "Sympathetic Nervous System"[Mesh]  #2 parasympath* OR sympath* OR sympathovagal OR vagal OR vagus OR "autonom* nerv*" OR "ANS" OR "PNS" OR "SNS"  #3 "heart rate variability" OR "cycle length variability" OR "R–R variability" OR "HRV" OR "heart rate recovery"  #4 ("Polycystic Ovary Syndrome"[Mesh] OR "polycystic ovary* disease*"[Title/Abstract] OR "PCOS"[Title/Abstract])  #5 #1 OR #2 OR #3  #6 #5 AND #4 |
| **Scopus** | #1 TITLE-ABS-KEY ( parasympath* OR sympath* OR sympathovagal OR vagal OR vagus OR "autonom* nerv*" OR "ans" OR "pns" OR "sns" )  #2 ALL ( "heart rate variability" OR "cycle length variability" OR "r--r variability" OR "hrv" OR "heart rate recovery" )  #3 TITLE-ABS-KEY ( "polycystic ovar* syndrome" OR "polycystic ovar* disease" OR "stein leventhal syndrome" OR "pcos" OR "sclerocystic ovar*" )  #4 #1 OR #2  #5 #3 AND #4  ( ( TITLE-ABS-KEY ( parasympath* OR sympath* OR sympathovagal OR vagal OR vagus OR "autonom* nerv*" OR "ans" OR "pns" OR "sns" ) ) OR ( ALL ( "heart rate variability" OR "cycle length variability" OR "r--r variability" OR "hrv" OR "heart rate recovery" ) ) ) AND ( TITLE-ABS-KEY ( "polycystic ovar* syndrome" OR "polycystic ovar* disease" OR "stein leventhal syndrome" OR "pcos" OR "sclerocystic ovar*" ) ) |
| **Cochrane** | ID Search Hits  #1 MeSH descriptor: [Autonomic Nervous System] explode all trees    #2 MeSH descriptor: [Sympathetic Nervous System] explode all trees    #3 MeSH descriptor: [Parasympathetic Nervous System] explode all trees  #4 MeSH descriptor: [Polycystic Ovary Syndrome] explode all trees    #5 (parasympath* OR sympath* OR sympathovagal OR vagal OR vagus OR "autonom* nerv*" OR "ans" OR "pns" OR "sns"):ti,ab,kw (Word variations have been searched)  #6 ("heart rate variability" OR "cycle length variability" OR "r--r variability" OR "hrv" OR "heart rate recovery") (Word variations have been searched)    #7 #1 OR #2 OR #3 OR #5 OR #6    #8 #7 AND #4 |
