# Supplementary material for: Exploring heart rate variability in polycystic ovary syndrome: implications for cardiovascular health: a systematic review and meta-analysis
Source: Syst Rev. 2024 Jul 24;13:194. doi: 10.1186/s13643-024-02617-x (PMC11271026; doi:10.1186/s13643-024-02617-x)
Supplement: Supplementary file 3 — Additional file 3: S3-document. Table of excluded studies during final screening. [file 13643_2024_2617_MOESM3_ESM.docx]

| **Author’s Name, Year of publication** | **Name of the Article** | **Reason of Exclusion** |
| --- | --- | --- |
| Giallauria et al(1), 2009 | Relationship between heart rate recovery and inflammatory markers in patients with polycystic ovary syndrome: a cross-sectional study | HRV measures were not reported |
| Ozcan Dag et al(2). 2015 | Autonomic dysfunction in patients with polycystic ovary syndrome | This article assessed autonomic dysfunction with means of a method other than HRV |
| De Sa et al(3). 2011 | Analysis of heart rate variability in polycystic ovary syndrome | This article did not report the raw data of HRV measures. The results were presented as box plots. We emailed the corresponded author to request for data. No response received. |
| Li et al(4). 2014 | Association of sympathetic nervous system activity with polycystic ovarian syndrome | Review article |
| Giallauria et al(5), 2008 | Abnormal heart rate recovery after maximal cardiopulmonary exercise stress testing in young overweight women with polycystic ovary syndrome | HRV measures were not reported |
| Thomson et al(6), 2010 | Heart rate recovery improves after weight loss in overweight and obese women with polycystic ovary syndrome | HRV measures were not reported |
| Graff et al(7), 2017 | Saturated Fat Intake Is Related to Heart  Rate Variability in Women with Polycystic  Ovary Syndrome | HRV measures were analyzed based on saturated fatty acid (SFA) level in PCOS cases and control group. |
| De Sa et al(8), 2013 | Heart rate variability as a method of assessing the autonomic nervous system in polycystic ovary syndrome | Narrative review article |
| Davis et al(9), 2018 | Correlation Between Physiologic and Osteopathic Measures of Sympathetic Activity in Women With Polycystic Ovary Syndrome | No control group without PCOS were involved and matched with PCOS cases. |
| Ramadoss et al(10), 2016 | Heart Rate Changes in Electroacupuncture Treated Polycystic Ovary in Rats | Study was conducted on rat samples |
| Kaya et al(11), 2009 | C-reactive protein and homocysteine levels are  associated with abnormal heart rate recovery in women with polycystic ovary syndrome | HRV measures were not reported |
| S´a et al(12), 2015 | Aerobic exercise improves cardiac autonomic modulation in women with  polycystic ovary syndrome | No control group without PCOS were involved and matched with PCOS cases. |
| Zheng et al(13), 2022 | Association Between Circulating Adiponectin and Heart Rate Recovery in Women With Polycystic Ovarian Syndrome | HRV measures were not reported |
| Pattnaik et al(14), 2021 | Comparison of autonomic function tests and high-sensitivity C-reactivity protein in overweight patients of polycystic ovarian syndrome and overweight controls | HRV measures were not reported |
| Dog et al(15), 2015 | Autonomic dysfunction in patients with polycystic ovary syndrome | Different HRV parameter was used |
| Ollila et al(16), 2019 | Effect of polycystic ovary syndrome on cardiac autonomic function at a late fertile age: a prospective Northern Finland Birth Cohort 1966 study | Control group was not properly matched with PCOS cases in terms of sample size and baseline characteristics (Cohort design). |

1. Giallauria F, Orio F, Lombardi G, Colao A, Vigorito C, Tafuri MG, et al. Relationship between heart rate recovery and inflammatory markers in patients with polycystic ovary syndrome: a cross-sectional study. Journal of Ovarian Research. 2009;2(1):1-7.

2. Dag ZO, Alpua M, Turkel Y, Isik Y. Autonomic dysfunction in patients with polycystic ovary syndrome. Taiwanese journal of obstetrics & gynecology. 2015;54(4):381-4.

3. de Sá JC, Costa EC, da Silva E, Zuttin RS, da Silva EP, Lemos TM, et al. Analysis of heart rate variability in polycystic ovary syndrome. Gynecological endocrinology : the official journal of the International Society of Gynecological Endocrinology. 2011;27(6):443-7.

4. Li W, Chen Y, Xu L. Association of sympathetic nervous system activity with polycystic ovarian syndrome. Clinical and experimental obstetrics & gynecology. 2014;41(5):499-506.

5. Giallauria F, Palomba S, Manguso F, Vitelli A, Maresca L, Tafuri D, et al. Abnormal heart rate recovery after maximal cardiopulmonary exercise stress testing in young overweight women with polycystic ovary syndrome. Clinical endocrinology. 2008;68(1):88-93.

6. Thomson RL, Buckley JD, Noakes M, Clifton PM, Norman RJ, Brinkworth GD. Heart rate recovery improves after weight loss in overweight and obese women with polycystic ovary syndrome. Fertility and sterility. 2010;93(4):1173-8.

7. Graff SK, Mario FM, Magalhaes JA, Moraes RS, Spritzer PM. Saturated fat intake is related to heart rate variability in women with polycystic ovary syndrome. Annals of Nutrition and Metabolism. 2017;71(3-4):224-33.

8. Sá JCFd, Costa EC, Silva Ed, Azevedo GD. Heart rate variability as a method of assessing the autonomic nervous system in polycystic ovary syndrome. Revista Brasileira de Ginecologia e Obstetrícia. 2013;35:421-6.

9. Davis SE, Hendryx J, Bouwer S, Menezes C, Menezes H, Patel V, et al. Correlation between physiologic and osteopathic measures of sympathetic activity in women with polycystic ovary syndrome. Journal of Osteopathic Medicine. 2019;119(1):7-17.

10. Ramadoss M, Ramanathan G, Subbiah AJ, Natrajan C. Heart Rate changes in electroacupuncture treated polycystic ovary in rats. Journal of clinical and diagnostic research: JCDR. 2016;10(3):CF01.

11. Kaya C, Akgül E, Pabuccu R. C-reactive protein and homocysteine levels are associated with abnormal heart rate recovery in women with polycystic ovary syndrome. Fertility and sterility. 2010;94(1):230-5.

12. Sá JC, Costa EC, Da Silva E, Tamburús NY, Porta A, Medeiros LF, et al. Aerobic exercise improves cardiac autonomic modulation in women with polycystic ovary syndrome. International journal of cardiology. 2016;202:356-61.

13. Sun W, Liu G, Liu B. Association between Circulating Adiponectin and Heart Rate Recovery in Women with Polycystic Ovarian Syndrome. Endocrine Research. 2022;47(2):56-63.

14. Pattnaik S, Gupta S, Saxena U, Matlani M, Kapoor R. Comparison of autonomic function tests and high-sensitivity C-reactivity protein in overweight patients of polycystic ovarian syndrome and overweight controls. Indian Journal of Physiology and Pharmacology. 2021;64(4):303-8.

15. Dag ZO, Alpua M, Turkel Y, Isik Y. Autonomic dysfunction in patients with polycystic ovary syndrome. Taiwanese Journal of Obstetrics and Gynecology. 2015;54(4):381-4.

16. Ollila M-M, Kiviniemi A, Stener-Victorin E, Tulppo M, Puukka K, Tapanainen J, et al. Effect of polycystic ovary syndrome on cardiac autonomic function at a late fertile age: a prospective Northern Finland Birth Cohort 1966 study. BMJ open. 2019;9(12):e033780.
