# Supplementary material for: Exploring heart rate variability in polycystic ovary syndrome: implications for cardiovascular health: a systematic review and meta-analysis
Source: Syst Rev. 2024 Jul 24;13:194. doi: 10.1186/s13643-024-02617-x (PMC11271026; doi:10.1186/s13643-024-02617-x)
Supplement: Supplementary file 4 — Additional file 4: S4-document. Forest plots of meta-analysis. [file 13643_2024_2617_MOESM4_ESM.docx]

**LF**

**A**

**B**

**C**

Forest plot graphs of LF analysis. **Panel A** indicates the comparison of LF parameter between women with PCOS and women in the control group. **Panel B** illustrates a subgroup analysis based on BMI (normal, overweight, and obese) regarding the LF and **Panel C** illustrates a subgroup analysis based on study design (case-control and cross-sectional) regarding the LF between women with PCOS and the control group. A random-effects model was used in this meta-analysis (I2>50), and standerized mean difference (SMD) was used to measure the effect size. A positive effect indicates higher values in women with PCOS than in women in the control group and vice versa. CI= Confidence Interval; SD= Standard Deviation; PCOS= Polycystic Ovarian Syndrome

**HF**

**A**

**B**

**C**

Forest plot graphs of HF analysis. **Panel A** indicates the comparison of HF parameter between women with PCOS and women in the control group. **Panel B** illustrates a subgroup analysis based on BMI (normal, overweight, and obese) regarding the HF and **Panel C** illustrates a subgroup analysis based on study design (case-control and cross-sectional) regarding the HF between women with PCOS and the control group. A random-effects model was used in this meta-analysis (I2>50), and standerized mean difference (SMD) was used to measure the effect size. A positive effect indicates higher values in women with PCOS than in women in the control group and vice versa. CI= Confidence Interval; SD= Standard Deviation; PCOS= Polycystic Ovarian Syndrome

**HFnu**

**A**

**B**

**C**

Forest plot graphs of HFnu analysis. **Panel A** indicates the comparison of HFnu parameter between women with PCOS and women in the control group. **Panel B** illustrates a subgroup analysis based on BMI (normal, overweight, and obese) regarding the HFnu and **Panel C** illustrates a subgroup analysis based on study design (case-control and cross-sectional) regarding the HFnu between women with PCOS and the control group. A random-effects model was used in this meta-analysis (I2>50), and standerized mean difference (SMD) was used to measure the effect size. A positive effect indicates higher values in women with PCOS than in women in the control group and vice versa. CI= Confidence Interval; SD= Standard Deviation; PCOS= Polycystic Ovarian Syndrome

**LFnu**

**A**

**B**

**C**

Forest plot graphs of LFnu analysis. **Panel A** indicates the comparison of LFnu parameter between women with PCOS and women in the control group. **Panel B** illustrates a subgroup analysis based on BMI (normal, overweight, and obese) regarding the LFnu and **Panel C** illustrates a subgroup analysis based on study design (case-control and cross-sectional) regarding the LFnu between women with PCOS and the control group. A random-effects model was used in this meta-analysis (I2>50), and standerized mean difference (SMD) was used to measure the effect size. A positive effect indicates higher values in women with PCOS than in women in the control group and vice versa. CI= Confidence Interval; SD= Standard Deviation; PCOS= Polycystic Ovarian Syndrome

**LF/HF ratio**

**A**

**B**

**C**

Forest plot graphs of LF/HF ratio analysis. **Panel A** indicates the comparison of LF/HF ratio parameter between women with PCOS and women in the control group. **Panel B** illustrates a subgroup analysis based on BMI (normal, overweight, and obese) regarding the LF/HF ratio and **Panel C** illustrates a subgroup analysis based on study design (case-control and cross-sectional) regarding the LF/HF ratio between women with PCOS and the control group. A random-effects model was used in this meta-analysis (I2>50), and standerized mean difference (SMD) was used to measure the effect size. A positive effect indicates higher values in women with PCOS than in women in the control group and vice versa. CI= Confidence Interval; SD= Standard Deviation; PCOS= polycystic ovarian syndrome

**TP**

**A**

**B**

**C**

Forest plot graphs of TP analysis. **Panel A** indicates the comparison of TP parameter between women with PCOS and women in the control group. **Panel B** illustrates a subgroup analysis based on BMI (normal, overweight, and obese) regarding the TP and **Panel C** illustrates a subgroup analysis based on study design (case-control and cross-sectional) regarding the TP between women with PCOS and the control group. A random-effects model was used in this meta-analysis (I2>50), and standerized mean difference (SMD) was used to measure the effect size. A positive effect indicates higher values in women with PCOS than in women in the control group and vice versa. CI= Confidence Interval; SD= Standard Deviation; PCOS= polycystic ovarian syndrome

**SDNN**

**A**

**B**

**C**

Forest plot graphs of SDNN analysis. **Panel A** indicates the comparison of SDNN parameter between women with PCOS and women in the control group. **Panel B** illustrates a subgroup analysis based on BMI (normal, overweight, and obese) regarding the SDNN and **Panel C** illustrates a subgroup analysis based on study design (case-control and cross-sectional) regarding the SDNN between women with PCOS and the control group. A random-effects model was used in this meta-analysis (I2>50), and standerized mean difference (SMD) was used to measure the effect size. A positive effect indicates higher values in women with PCOS than in women in the control group and vice versa. CI= Confidence Interval; SD= Standard Deviation; PCOS= polycystic ovarian syndrome.

**PNN50**

**A**

**B**

**C**

Forest plot graphs of PNN50 analysis. **Panel A** indicates the comparison of PNN50 parameter between women with PCOS and women in the control group. **Panel B** illustrates a subgroup analysis based on BMI (normal, overweight, and obese) regarding the PNN50 and **Panel C** illustrates a subgroup analysis based on study design (case-control and cross-sectional) regarding the PNN50 between women with PCOS and the control group. A random-effects model was used in this meta-analysis (I2>50), and standerized mean difference (SMD) was used to measure the effect size. A positive effect indicates higher values in women with PCOS than in women in the control group and vice versa. CI= Confidence Interval; SD= Standard Deviation; PCOS= polycystic ovarian syndrome

**NN50**

Forest plot graph of NN50 analysis. It indicates the comparison of NN50 parameter between women with PCOS and women in the control group. A fixed-effect model was used in this meta-analysis, and standerized mean difference (SMD) was used to measure the effect size. A positive effect indicates higher values in women with PCOS than in women in the control group and vice versa. CI= Confidence Interval; SD= Standard Deviation; PCOS= polycystic ovarian syndrome

**SDANN**

Forest plot graph of SDANN analysis. It indicates the comparison of SDANN parameter between women with PCOS and women in the control group (I2<50).A positive effect indicates higher values in women with PCOS than in women in the control group and vice versa. CI= Confidence Interval; SD= Standard Deviation; PCOS= Polycystic Ovarian Syndrome

**Mean-RR**

**A**

**B**

**C**

Forest plot graphs of Mean R-R analysis. **Panel A** indicates the comparison of Mean R-R parameter between women with PCOS and women in the control group. **Panel B** illustrates a subgroup analysis based on BMI (normal, overweight, and obese) regarding the Mean R-R and **Panel C** illustrates a subgroup analysis based on study design (case-control and cross-sectional) regarding the Mean R-R between women with PCOS and the control group. A random-effects model was used in this meta-analysis(I2>50), and standerized mean difference (SMD) was used to measure the effect size. A positive effect indicates higher values in women with PCOS than in women in the control group and vice versa. CI= Confidence Interval; SD= Standard Deviation; PCOS= polycystic ovarian syndrome

**RMSSD**

**A**

**B**

**C**

Forest plot graphs of RMSSD analysis. **Panel A** indicates the comparison of RMSSD parameter between women with PCOS and women in the control group. **Panel B** illustrates a subgroup analysis based on BMI (normal, overweight, and obese) regarding the RMSSD and **Panel C** illustrates a subgroup analysis based on study design (case-control and cross-sectional) regarding the RMSSD between women with PCOS and the control group. A random-effects model was used in this meta-analysis (I2>50), and SMD was used to measure the effect size. A positive effect indicates higher values in women with PCOS than in women in the control group and vice versa. CI= Confidence Interval; SD= Standard Deviation; PCOS= polycystic ovarian syndrome; SMD= standardized mean difference.
