# Supplementary material for: Exploring heart rate variability in polycystic ovary syndrome: implications for cardiovascular health: a systematic review and meta-analysis
Source: Syst Rev. 2024 Jul 24;13:194. doi: 10.1186/s13643-024-02617-x (PMC11271026; doi:10.1186/s13643-024-02617-x)
Supplement: Supplementary file 5 — Additional file 5: High resolution funnel plots. [file 13643_2024_2617_MOESM5_ESM.docx]

**RMSSD**

Mean-RR

SDNN

SDANN

**PNN50**

NN50

LF/HF ratio

HFnu

LFnu

HF

LF

TP
