# Supplementary material for: Exploring heart rate variability in polycystic ovary syndrome: implications for cardiovascular health: a systematic review and meta-analysis
Source: Syst Rev. 2024 Jul 24;13:194. doi: 10.1186/s13643-024-02617-x (PMC11271026; doi:10.1186/s13643-024-02617-x)
Supplement: Supplementary file 6 — Additional file 6: High resolution meta-regression plots. [file 13643_2024_2617_MOESM6_ESM.docx]

HFnu

BHR

BMI

SBP

LFnu

BMI

SBP

BHR

LF/HF

HF

SDNN
