# Supplementary material for: Exploring heart rate variability in polycystic ovary syndrome: implications for cardiovascular health: a systematic review and meta-analysis
Source: Syst Rev. 2024 Jul 24;13:194. doi: 10.1186/s13643-024-02617-x (PMC11271026; doi:10.1186/s13643-024-02617-x)
Supplement: Supplementary file 7 — Additional file 7: High resolution sensitivity analysis forest plots. [file 13643_2024_2617_MOESM7_ESM.docx]

**TP**

Forest plot of sensitivity analysis with one-study removed approach for TP parameter between women with PCOS and women in the control group. The sensitivity analysis results align closely with the primary analysis results, indicating robustness in the findings. CI= Confidence Interval, PCOS= polycystic ovarian syndrome.

**LFnu**

Forest plot of sensitivity analysis with one-study removed approach for LFnu parameter between women with PCOS and women in the control group. The sensitivity analysis results align closely with the primary analysis results, indicating robustness in the findings. CI= Confidence Interval, PCOS= Polycystic Ovarian Syndrome.

**HFnu**

Forest plot of sensitivity analysis with one-study removed approach for HFnu parameter between women with PCOS and women in the control group. The sensitivity analysis results align closely with the primary analysis results, indicating robustness in the findings. CI= Confidence Interval, PCOS= Polycystic Ovarian Syndrome.

**LF/HF ratio**

Forest plot of sensitivity analysis with one-study removed approach for LF/HF ratio parameter between women with PCOS and women in the control group. The sensitivity analysis results align closely with the primary analysis results, indicating robustness in the findings. CI= Confidence Interval, PCOS= polycystic ovarian syndrome.

**HF**

Forest plot of sensitivity analysis with one-study removed approach for HF parameter between women with PCOS and women in the control group. The sensitivity analysis results align closely with the primary analysis results, indicating robustness in the findings. CI= Confidence Interval, PCOS= polycystic ovarian syndrome.

**LF**

Forest plot of sensitivity analysis with one-study removed approach for LF parameter between women with PCOS and women in the control group. The sensitivity analysis results align closely with the primary analysis results, indicating robustness in the findings. CI= Confidence Interval, PCOS= polycystic ovarian syndrome.

**SDNN**

Forest plot of sensitivity analysis with one-study removed approach for SDNN parameter between women with PCOS and women in the control group. The sensitivity analysis results align closely with the primary analysis results, indicating robustness in the findings. CI= Confidence Interval, PCOS= polycystic ovarian syndrome.

**PNN50**

Forest plot of sensitivity analysis with one-study removed approach for PNN50 parameter between women with PCOS and women in the control group. The sensitivity analysis results align closely with the primary analysis results, indicating robustness in the findings. CI= Confidence Interval, PCOS= polycystic ovarian syndrome.

**NN50**

Forest plot of sensitivity analysis with one-study removed approach for NN50 parameter between women with PCOS and women in the control group. The sensitivity analysis results align closely with the primary analysis results, indicating robustness in the findings. CI= Confidence Interval, PCOS= polycystic ovarian syndrome.

**SDANN**

Forest plot of sensitivity analysis with one-study removed approach for SDANN parameter between women with PCOS and women in the control group. The sensitivity analysis results align closely with the primary analysis results, indicating robustness in the findings. CI= Confidence Interval, PCOS= polycystic ovarian syndrome.

**Mean-RR**

Forest plot of sensitivity analysis with one-study removed approach for Mean-RR parameter between women with PCOS and women in the control group. The sensitivity analysis results align closely with the primary analysis results, indicating robustness in the findings. CI= Confidence Interval, PCOS= polycystic ovarian syndrome.

**RMSSD**

Forest plot of sensitivity analysis with one-study removed approach for RMSSD parameter between women with PCOS and women in the control group. The sensitivity analysis results align closely with the primary analysis results, indicating robustness in the findings. CI= Confidence Interval, PCOS= polycystic ovarian syndrome.
